# Anomalous Origin of the Left Main Coronary Artery from the Right Coronary Sinus

**DOI:** 10.7759/cureus.80995

**Published:** 2025-03-22

**Authors:** Devesh Kumar, Kartik Aggarwal, Saurav Suman, Prerna Garg, Satyavir Yadav

**Affiliations:** 1 Cardiology, Vardhman Mahavir Medical College and Safdarjung Hospital, New Delhi, IND; 2 Cardiology, All India Institute of Medical Sciences, New Delhi, New Delhi, IND

**Keywords:** anomalous coronary artery origin, computed tomography coronary angiography (ctca), high risk anatomy, ischemia, refractory angina, reimplantation of the left main coronary

## Abstract

An elderly gentleman in his 60s presented with angina on exertion and dyspnea on exertion (NYHA class 3). On evaluation, he was found to have significant obstructive triple vessel disease. He also had an anomalous origin of the left main from the right coronary sinus with a long and tortuous left main coronary artery, which was seen to be running anterior to the pulmonary artery. He was treated with coronary artery bypass grafting with three grafts and re-implantation of the left main coronary artery to the left coronary sinus and was discharged with no postoperative complaints. This case report highlights a unique presentation of coronary artery anomaly in an elderly patient and underscores the importance of timely diagnosis and intervention.

## Introduction

Coronary artery anomalies (CAAs) are characterized by abnormal origin or course or termination of any of the three main epicardial arteries and are seen in 0.6-1.3% of the general population [1]. CAAs are clinically significant as they are associated with sudden cardiac death (SCD), especially in the younger population engaged in sports [2]. An autopsy study showed that anomalous aortic origin of a coronary artery was the second most common cause of SCD in young athletes [3]. With the widespread use of invasive and non-invasive coronary imaging, CAAs are now most commonly discovered as an incidental finding during the diagnostic workup for coronary artery disease. They, however, are significant when associated with angina, inducible ischemia, or high-risk features and hence warrant stress testing and delineation of anatomy whenever detected incidentally.

## Case presentation

An elderly gentleman in his 60s, a known hypertensive for the last 10 years, presented to our outpatient department with complaints of exertional angina and dyspnea for the last six months, which was NYHA class II initially but had worsened to NYHA class III over the last two months. He was treated with guideline-directed medical therapy (aspirin, high-dose statin, beta-blocker, angiotensin-converting enzyme inhibitor, mineralocorticoid receptor antagonist, sodium glucose co-transporter 2 inhibitors, and diuretics) at a private hospital, but due to worsening of symptoms, he was referred to our center. On examination, his vitals were normal, and there were no significant findings on cardiac examinations.

**Figure 1:**
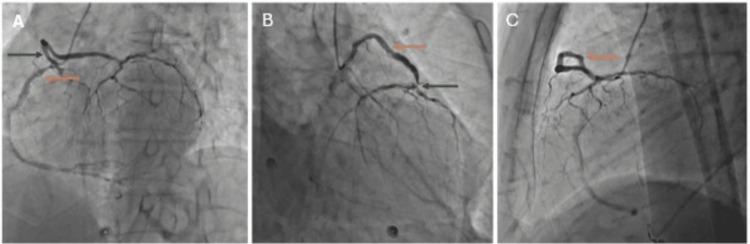
Various angiography views with anomalous origin of the left main coronary artery from the right coronary sinus (A) Filling of the left (green arrow) and right (orange arrow) coronary arteries with single injection in the right coronary sinus in the left anterior oblique cranial view using a 5French Tiger catheter view with significant disease in the right coronary artery at both the ostia and crux. (B) Selective cannulation of the left main coronary artery (orange arrow) from the right coronary sinus with a significantly long left main coronary artery and significant disease at the bifurcation (green arrow) into both the left anterior descending and the left circumflex artery, with both these vessels being significantly diseased. (C) Simultaneous filling of the left coronary (orange arrow) and right coronary system with injection in the right coronary sinus, with the left main coronary artery taking a circumferential route and running anterior to the pulmonary artery.

**Figure 2:**
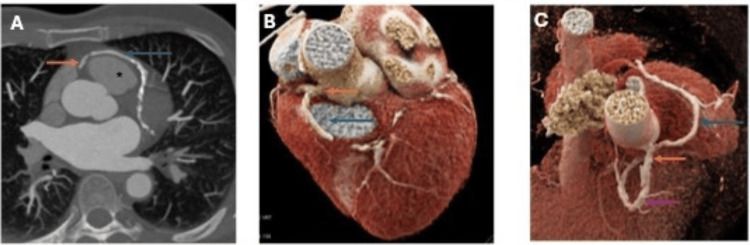
(A) Axial thick sections showing the left main coronary artery (blue arrow) arising from the right coronary sinus (orange arrow) and passing anterior to the pulmonary artery (*). (B and C) Volume-rendered CT images with the left main artery (blue arrow) arising from the right coronary sinus (orange arrow). Distal right coronary artery is also seen (purple arrow).

His 2D transthoracic echocardiography revealed moderate left ventricular systolic dysfunction with an ejection fraction of 35% and hypokinesia in the anterior wall, anteroseptal wall, and anterolateral wall at the basal, mid, and apical levels of the left ventricle. He was taken up for coronary angiography (Figure 1), which revealed significant stenosis (maximum stenosis of 90%) in the right coronary artery in the osteo-proximal segment and significant stenosis at the crux (maximum stenosis of 70%). The left main coronary artery (LMCA) had an anomalous origin from the right coronary sinus (opposite sinus) and was longer than its usual length with significant (80%) obstruction in the left anterior descending artery and diffuse disease in the left circumflex artery with maximum stenosis of 80%. Since our patient suffered from refractory angina despite maximal medical therapy and triple vessel disease with an abnormal left main arising from the right coronary sinus, he mandated revascularization with coronary artery bypass grafting (CABG). Furthermore, the patient underwent a computed tomography coronary angiography (CTCA) (Figure 2), which revealed a long LMCA with origin from the right coronary sinus and running anterior to the pulmonary artery.

The patient was subsequently referred for CABG, with grafts to all three vessels and reimplantation of the LMCA into the left coronary sinus. Following surgery, the patient recovered well, remained hemodynamically stable, and was discharged home. On follow-up after three months, the patient had an improved left ventricular ejection fraction of 45-50%. He was NYHA class 1 and was able to walk 5 miles at a steady pace without much difficulty.

## Discussion

**Figure 3:**
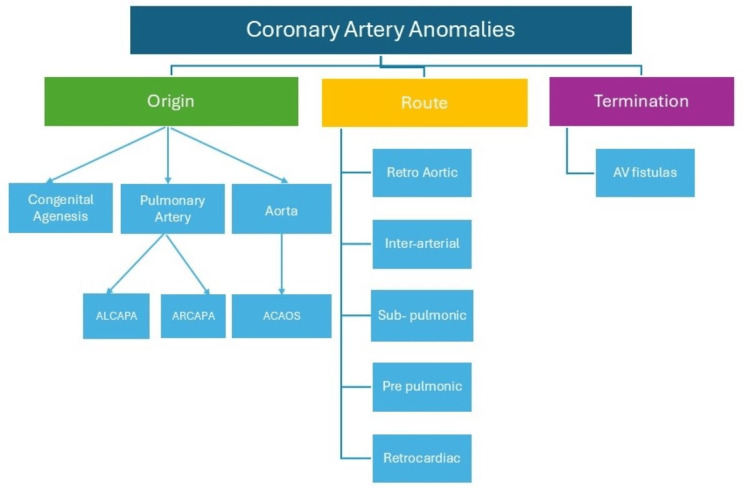
Classification of coronary artery anomalies ALCAPA, anomalous left coronary artery from the pulmonary artery; ARCAPA, anomalous right coronary artery from the pulmonary artery; ACAOS, anomalous coronary artery from opposite sinus Figure illustrated by Devesh Kumar

CAAs, a group of congenital conditions characterized by abnormal origin or course of any of the three main epicardial arteries, are known to be associated with SCD, especially in young athletes [1-2]. In a large autopsy study, the anomalous aortic origin of a coronary artery was the second most common cause of SCD in young athletes [3]. With the widespread use of non-invasive coronary imaging, CAAs are now most commonly discovered incidentally during the diagnostic workup for coronary artery disease. CAAs can be classified based on origin, course, and termination (Figure 3).

Anomalous origin may be either from the pulmonary artery (0.01%) or aorta (0.1%) [4]. While anomalous origin from the pulmonary artery of both the left and right coronary systems is ominous, the left main coronary origin from the pulmonary artery is far more concerning than the right. Anomalous origin of the LMCA from the pulmonary artery (ALCAPA) presents in 90% of the patients during their infancy and in 10% during adulthood [5]. While in infancy it presents with massive anterolateral myocardial infarction and heart failure, in adults it may have a more variable clinical presentation varying from SCD to incidentally diagnosed cases. Anomalous origin of the right coronary artery from the pulmonary artery (ARCAPA), on the other hand, presents later with angina or is diagnosed incidentally and is considered less concerning than its counterpart. However, given its predisposition to produce ischemia and SCD, it mandates surgical correction [6]. Another rare form of anomalous origin is the complete atresia of LMCA ostium, where it is replaced by a fibrous tract and produces clinically worrisome angina [7].

The anomalous origin of coronary from the aorta may be from any of the sinuses - the opposite sinus or the non-coronary sinus. The non-coronary sinus origin of coronary is the rarest of the three. The most common anomaly is origin of the LMCA arising from the right sinus. The left main coronary arising from the right sinus is more common. The LMCA has a longer course, which predisposes it to more hemodynamic strain than the right one [8]. The presence of other high-risk anatomic features determines if the anomalous origin of the artery would have clinical implications such as inter-arterial course, slitlike orifice, high takeoff (high origin of the coronary of more than 1cm from the sinotubular junction), and acute angle takeoff (<45 degree) of the proximal coronary artery from the aorta [9]. These high-risk anatomic features predispose to ischemic complications and subsequently may lead to SCD.

The anomalous course of the coronaries is further subdivided into five subtypes, namely, retro-aortic, inter-arterial, sub-pulmonic, pre-pulmonic, and retro-cardiac in the order of their occurrence. Of these, the inter-arterial subtype is the most concerning, as the anomalous artery runs between the aorta and pulmonary artery and consequently has the highest predisposition of getting compressed especially during strenuous activities. The retro-aortic course is the most common, and the retrocardiac course is the least common. This inter-arterial course is most often present concomitantly with other high-risk anatomic features, thus predisposing the patients to a high risk of SCD [6].

The anomalous termination of the coronary artery may happen in any of the chambers, and these are generally known as coronary arteriovenous fistulas. These are generally innocent bystanders and need to be treated only if they have a significant shunt of more than 1.5:1 and produce clinically significant ischemia and/or left ventricular volume overload. They are generally non-ominous not warranting any treatment.

While most coronary anomalies are diagnosed incidentally on invasive coronary angiography during the evaluation of coronary artery disease, CTCA continues to be the gold standard. CTCA involves lesser radiation, has higher 3D visualization of the coronary arteries with better anatomic correlation of the coronaries with the surrounding structures, is more readily available, and can optimally visualize the high-risk features, thus making it the preferred modality for diagnosis [10]. Coronary angiography, although unique, is invasive, has higher radiation exposure, and fails to delineate the high-risk anatomic features and relationship of the coronary to the adjoining cardiac structures [11]. Cardiac MRI has evolved as an equally valuable tool, but the lesser availability, higher cost, and lack of trained professionals have prevented it from becoming the first-line diagnostic investigation [12].

All patients irrespective of symptoms should be screened for ischemia with stress imaging. The reproduction of ischemia in the territory of an anomalous coronary artery is the closest predictor of cardiac events. While the absence of ischemia does not preclude the patient from having future cardiac events, the presence of reproducible ischemia in the presence of symptoms is the strongest predictor of future cardiac events and SCD. Exercise treadmill tests being physiologic are often the first-line investigation, but given the low sensitivity and specificity, nuclear tests using thallium or dobutamine echo may be preferred for demonstrating ischemia [13]. To summarize, all the data from the current literature suggests that the presence of anginal symptoms combined with the reproduction of ischemia in the territory of the anomalous coronary artery is the strongest predictor of future cardiac events and thus forms the strongest indication for surgery.

After diagnosis, management of patients with CAAs varies based on the anomaly. We will first discuss the management of patients with anomalous origin from the pulmonary artery and subsequently deal with the anomalous coronary artery from the opposite sinus. Patients with anomalous origin of the coronary artery from the pulmonary artery warrant surgical repair with restoration of dual coronary artery system [14]. This is most frequently done by re-implantation of the coronary into the ascending aorta; however, in cases where the ostia is very distant from the aorta, creation of intra-pulmonary shunt may be done to connect the ostia of the anomalous artery to the ascending aorta (Takeuchi repair) [15]. Lastly, CABG remains an alternative treatment strategy when the above two options are not possible. In the largest cohort till date, operative mortality is around 6%, with no data on long-term follow-up [16].

**Figure 4:**
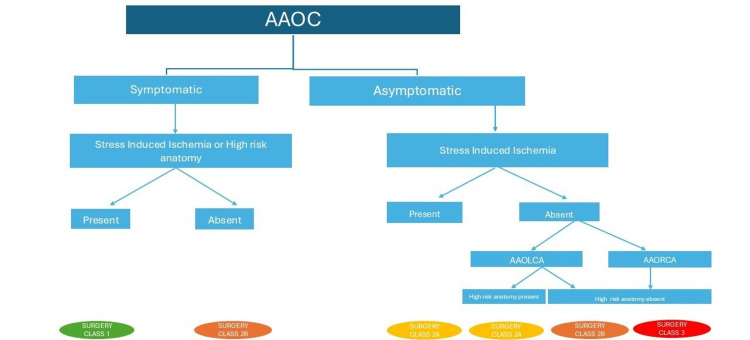
Approach and management of anomalous aortic origin of coronaries Simplified image showing a pragmatic approach to a patient with anomalous origin of coronary based on the symptom status, the presence of high-risk anatomy, and stress-induced ischemia. AAOC, anomalous aortic origin of a coronary artery; AAOLCA, anomalous aortic origin of the left coronary artery; AAORCA, anomalous aortic origin of the right coronary artery Figure illustrated by Devesh Kumar

Management decisions are more nuanced when dealing with anomalous origin from the opposite sinus, which generally requires surgical treatment in patients with symptoms and the presence of either inducible ischemia on non-invasive testing or the presence of high-risk anatomy (Figure 4).

In asymptomatic patients with stress-induced ischemia on non-invasive testing, surgery is again the preferred treatment. Patients with anomalous origin of LMCA from the right sinus with no inducible ischemia and no high-risk anatomy but age less than 35 years may still be considered for surgery. In patients with anomalous origin of the right coronary artery from the left coronary sinus with neither inducible ischemia nor high-risk anatomy, surgery is not advisable [17]. In patients with a small intra-mural course, direct reimplantation of the anomalous vessel in the correct aortic sinus and osteoplasty are the preferred techniques. In patients with a longer intramural course, unroofing is preferred, in which the common wall between the anomalous vessel and the aorta is resected and a neo-ostium is opened in the anatomically correct sinus [15]. Finally, CABG should be restricted to patients with significant atherosclerosis or when an alternative approach is not feasible. Hence, significant competitive flow has to be expected most of the time, undermining the long-term patency of the bypass conduit [18]. Patients with an atresia of LMCA often have a fibrous tract replacing the LMCA with significant ischemia and warrant surgical correction with CABG [10].

Most coronary arteriovenous fistulas are small and are thus asymptomatic. An invasive approach is needed only in cases with significant left-to-right shunt or coronary steal phenomena, resulting in myocardial ischemia and ventricular overload [19]. Choice of technique for fistula closure depends on fistula morphology, course, tortuosity, and the presence of an aneurysmal dilatation of the afferent vessel. Surgical ligation at the drainage site is indicated for complex fistulas showing an overall operative mortality of 2% [16]. Percutaneous closure with coils, covered stents, and vascular plugs is preferred in cases with a single non-tortuous fistula [19].

## Conclusions

Patients with refractory angina should be subjected to early imaging as the disease distribution and severity may be very concerning. It is imperative for us to be aware of the clinical significance of anomalous coronary artery origin in patients with angina irrespective of the age at presentation. In patients with anomalous origin of the coronary artery detected on angiography, CTCA is mandatory to delineate and further risk-stratify based on anatomic features. Such patients if asymptomatic should undergo stress imaging to rule out ischemia to decide the treatment plan. ALCAPA, whether symptomatic or not, warrants surgery. All patients with anomalous origin of the coronary artery should be screened for the presence of symptoms, high-risk anatomic features, and the presence of stress-induced ischemia as these are important parameters that help clinicians decide further management.
